# How vision governs the collective behaviour of dense cycling pelotons

**DOI:** 10.1098/rsif.2019.0197

**Published:** 2019-07-10

**Authors:** J. Belden, M. M. Mansoor, A. Hellum, S. R. Rahman, A. Meyer, C. Pease, J. Pacheco, S. Koziol, T. T. Truscott

**Affiliations:** 1 Naval Undersea Warfare Center, Newport, RI 02841, USA; 2 Department of Mechanical and Aerospace Engineering, Utah State University, Logan, UT 84322, USA; 3 Robbins College of Health and Human Sciences, Baylor University, Waco, TX 76798, USA; 4 VeloCam Services, New York, NY, USA; 5 CSAIL, Massachusetts Institute of Technology, Boston, MA 02139, USA; 6 School of Engineering and Computer Science, Baylor University, Waco, TX 76798, USA

**Keywords:** collective behaviour, cycling pelotons, visual sensory system, visual field, bicycling

## Abstract

In densely packed groups demonstrating collective behaviour, such as bird flocks, fish schools or packs of bicycle racers (cycling pelotons), information propagates over a network, with individuals sensing and reacting to stimuli over relatively short space and time scales. What remains elusive is a robust, mechanistic understanding of how sensory system properties affect interactions, information propagation and emergent behaviour. Here, we show through direct observation how the spatio-temporal limits of the human visual sensory system govern local interactions and set the network structure in large, dense collections of cyclists. We found that cyclists align in patterns within a ± 30° arc corresponding to the human near-peripheral visual field, in order to safely accommodate motion perturbations. Furthermore, the group structure changes near the end of the race, suggesting a narrowing of the used field of vision. This change is consistent with established theory in psychology linking increased physical exertion to the decreased field of perception. Our results show how vision, modulated by arousal-dependent neurological effects, sets the local arrangement of cyclists, the mechanisms of interaction and the implicit communication across the group. We furthermore describe information propagation phenomena with an analogous elastic solid mechanics model. We anticipate our mechanistic description will enable a more detailed understanding of the interaction principles for collective behaviour in a variety of animals.

## 1. Introduction

**Figure 1.**
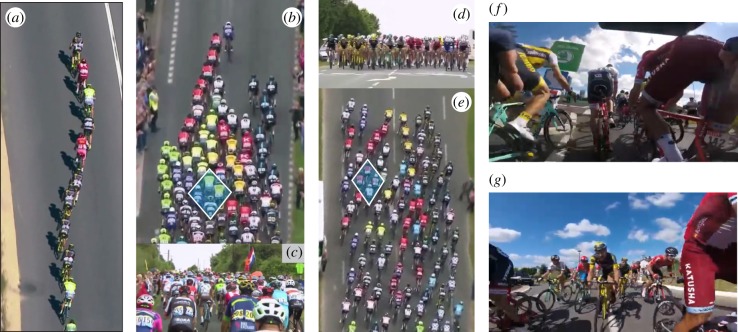
Pelotons take many formations in the professional TdF race. (*a*) In a *line*, cyclists follow one another closely to reduce aerodynamic drag. (*b*–*e*) More frequently, cyclists pack tightly in formations spanning the road with shapes such as (*b*) *arrow*, (*e*) *flat head* and others (see figure 2 and electronic supplementary material, figure S1). (*c*,*d*) Views from rear of (*b*) and front of (*e*), respectively. (*f*, *g*) The basic diamond pattern is evident from internal camera views (image credit: GoPro World), as well as from overhead views in (*b*,*e*). Image credits for (*a*–*e*): A.S.O. Eurosport, with permissions. (Online version in colour.)

Self-organized collective behaviour, employed by a range of species including birds [1–4], insects [5–8], fish [9–13] and even human crowds [14–17], is characterized by often remarkable global motion arising from local inter-individual interactions [18–20]. Collective behaviour in animals confers benefits related to foraging [21], predator evasion [22,23] and energy conservation [9,17,24,25]. In cycling pelotons, large groups of bicycle racers move in dense configurations to conserve energy through aerodynamic drafting (typical spacing ≪ bike length, typical speed ≈15 m s^−1^). Multi-day professional stage races such as the Tour de France (TdF) cover ≈3500 km in 21 days and feature a variety of emergent formations arising under different racing conditions as shown in figure 1 (see also electronic supplementary material, figure S1). The TdF includes individual goals, team objectives, terrain changes and other variables that result in a range of group dynamics playing out over different temporal and spatial scales [17]. However, the persistent feature is a densely packed peloton with classifiable global shapes that contains the bulk of the cyclists. Despite limited visibility within the peloton, collisions are rare even as motion perturbations routinely initiate waves that propagate through the group. The local principles that allow the group to move seamlessly as a whole, avoiding collisions while maintaining cohesion, also characterize other collective groups in nature [19,20].

In dense, moving animal groups, it is not clear whether individuals arrange themselves according to sensory function [2,26–28], optimal energetic benefit [9] or some combination thereof [20]. Moreover, our understanding of *how* sensor attributes affect group dynamics is still nascent [5,27–30]. Recently, it was shown that long-standing models of vision-based interaction (e.g. [2,31]) produce significantly different results when realistic assumptions about the visual sensory system are used as opposed to widely employed assumptions that oversimplify the visual system of the animal under consideration [30]. Yet, experimental data linking details of animal sensory systems to features of collective behaviour are sparse.

In cycling pelotons, the assumption has been that the internal structure follows from optimal drafting configuration [17], given that the drafting benefit in isolated pairs of cyclists is highly sensitive to relative positioning [32–34]. However, recent work has shown that the energetic benefit in the interior of a peloton is not particularly sensitive to local configuration [35]. We instead suggest that cyclist arrangement and local interaction principles are governed by details of the visual sensory systems. While factors such as strategy and terrain may affect cyclist positioning over longer time scales (e.g. minutes), we propose that sensory function shapes the moment-by-moment dynamics.

To test our hypothesis, we examine aerial television footage from stages of the 2016 TdF, and measure cyclist position, network structure and properties of information transfer, which is described herein as wave propagation. We provide evidence that these characteristics of the collective peloton arise from details of the human visual sensory system. The internal structure and information transfer behaviour are shown to change in conditions of high individual energetic output, which can be related to a change in sensor system function. Finally, we define an analogous elastic solid mechanical model that captures the properties of wave propagation within the peloton.

## 2. Observations and methods

The TdF is the premiere professional road cycling stage race and consists of more than 20 teams of eight riders competing for individual daily victories and overall lowest cumulative time after three weeks of racing. These opposing objectives create multiple dynamics within a given daily stage (see electronic supplementary material for more detail), but the majority of riders spend the day traversing in a tightly packed peloton, as shown in figure 1. The peloton can take on many forms depending on race conditions, terrain and team or individual objectives. These emergent global patterns are categorized into common persistent shapes, with the most prevalent being the echelon formation (see electronic supplementary material, figure S1). These formations are captured by helicopter for aerial television footage throughout the race, which we analyse here.

A series of image processing routines, described in more detail in the electronic supplementary material, is used to enable quantitative analysis down to the scale of the individual cyclists. Several variables are defined in the ensuing sections and these symbols are summarized in the electronic supplementary material, table S1. In each video clip, originally captured at 30 frames per second (fps) and lasting typically tens of seconds, we track the position of each cyclist in the sequence. Images and cyclist positions are then projected into a metric reference frame defined using known road marking lengths (electronic supplementary material, figure S2). From these transformed data, we can measure the distance Δ*s* and angle *θ* between neighbouring cyclists. Thus, our dataset contains quantitative individual and global information across a wide range of racing conditions, terrain and energetic output.

**Figure 2.**
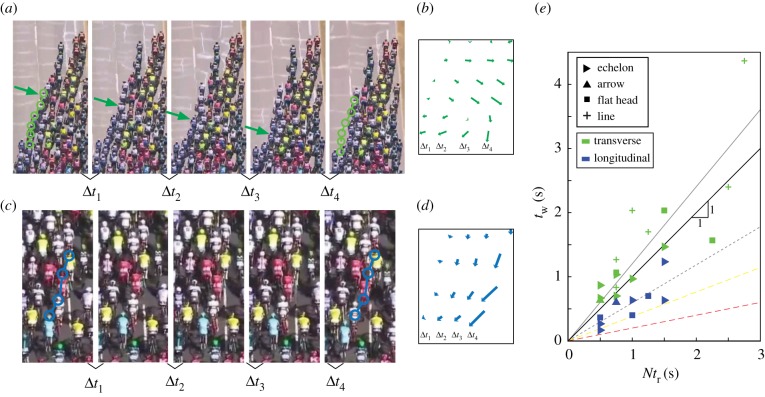
Two types of waves are observed to propagate through the peloton. (*a*) *Transverse* waves are characterized by cyclist motion perpendicular to the direction of peloton travel (see also electronic supplementary material, video S1, which shows this sequence); circles show the initial location of cyclists, arrows show the location of the wavefront. (*b*) Arrows indicate displacement of each of six riders affected by the transverse wave relative to a point fixed with respect to the moving peloton for the time instances shown in (*a*). (*c*) In *longitudinal* waves, the primary motion of affected cyclists is backward relative to the direction of peloton travel (see also electronic supplementary material, video S2); circles show the initial location of cyclists. (*d*) Displacement of four cyclists affected by the longitudinal wave relative to a point fixed with respect to the moving peloton for the time instances shown in (*c*). (*e*) For transverse waves, *t*_w_ ≈ *Nt*_r_ (best-fit line in grey has a slope of 1.2). Longitudinal waves propagate faster (grey dashed best-fit line is *t*_w_ = 0.6 *Nt*_r_). The lower two dashed lines are extrapolated from data for dunlin flocks [1] (yellow dashed line) and crowds of human sports fans performing the wave [14] (red dashed line). Image credits for (*a*,*c*): A.S.O. Eurosport, with permissions. (Online version in colour.)

Within the different global formations that emerge, we observe that cyclists consistently arrange themselves in a diamond-shaped lattice structure as shown in figure 1*b*,*e*. This alignment is confirmed by camera footage from within the peloton, figure 1*f*,*g*, which also indicates how restricted the field of vision is for an individual. In these dense arrangements, a perturbation in cyclist motion from the mean peloton heading has the potential to cause a crash, yet these catastrophic events are relatively rare. Rather, motion perturbations are seamlessly accommodated and typically result in waves that propagate through the group, as shown in figure 2 and electronic supplementary material, videos S1 and S2. Two modes of wave propagation are observed, which we label transverse and longitudinal, referring to the primary direction of the perturbed cyclist motion relative to the mean peloton motion. Transverse waves, figure 2*a*,*b*, are typically initiated by motion of a cyclist perpendicular to the forward direction of peloton travel, with trailing cyclists also moving laterally in sequence to preserve the network alignment. In longitudinal waves, the primary motion of affected cyclists is backward relative to the direction of peloton travel, as shown in figure 2*c*,*d*. This type of wave motion may be initiated by the sudden slowing of a cyclist, or by a rider moving backward through the peloton.

Waves are identified visually from processed image sequences of the helicopter television footage, which have been projected into a metric reference frame. The position of each wave-affected cyclist relative to the centroid of all cyclists is plotted for each frame in the sequence (electronic supplementary material, figure S7). These data combined with the visual inspection are used to determine the frame at which each affected rider first moves in response to the wave. The displacement of the wavefront relative to the instantaneous location of the first wave-affected rider is plotted against time and fit with a line to determine the wave speed *V*_*ϕ*_. The total time for the wave to propagate from the first to last affected cyclist is defined as *t*_w_. In addition to measuring the wave speed, the centre-to-centre distance Δ*s* between successive wave-affected cyclists is measured on the frame on which the wave is initiated, and the mean of this distance $\bar{\Delta s}$ is computed on this frame. In the next section, we use the properties of these waves to gain insight into the underlying interaction principles and their relationship to human vision.

## 3. Analysis and discussion

### 3.1. Wave propagation behaviour

For a range of peloton formations, we observe instances of transverse and longitudinal wave types and measure the total wave propagation time *t*_w_ as a function of the product of simple reaction time to visual stimuli (*t*_r_ = 250 ms [36]) and number of cyclists affected by the wave *N*, which is plotted in figure 2*e*. If each agent were responding to the visually detected motion of their nearest neighbour, we would expect *t*_w_ = *Nt*_r_, which is the trend followed by the transverse waves. The longitudinal waves, however, propagate faster than if cyclists are simply responding to their nearest neighbour. This type of behaviour has been observed in other groups including sporting event crowds [14] and dunlin flocks [1]. However, we are not aware of previous studies showing two different intrinsic time scales for wave propagation within a single collective group.

**Figure 3.**
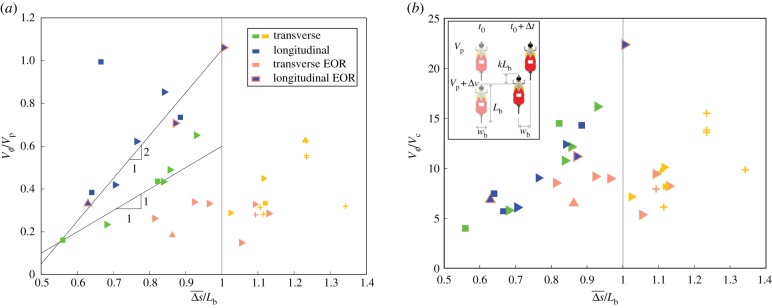
Waves demonstrate spacing-dependent speed. (*a*). Wave speed normalized by peloton velocity *V*_*ϕ*_/*V*_p_ as a function of the average normalized spacing between nearest neighbours $\bar{\Delta s}/L_{b}$; symbol shapes are the same as in figure 2*e*. The longitudinal wave data point that is far above the line corresponds to an uphill case, such that *V*_p_ is smaller than for a typical flat road case. The lines shown indicate that longitudinal waves propagate two times faster than transverse waves, which is consistent with the measured wave propagation times shown in figure 2*e*. (*b*). Using an alternate characteristic velocity *V*_c_ to normalize wave speed (where *V*_c_ = Δ*v* for longitudinal waves and *V*_c_ = *V*_trans_ for transverse waves) results in a collapse of the transverse and longitudinal data for $\bar{\Delta s}/L_{b}<1$, including the data point for the uphill case. In general, *V*_*ϕ*_/*V*_c_ is a linear function of $\bar{\Delta s}/L_{b}$. The exception to this trend is transverse waves occurring in the end of race (EOR) conditions for which *V*_*ϕ*_/*V*_c_ ≈ constant (pink symbols). For $\bar{\Delta s}/L_{b}>1$, we observe no longitudinal waves as the line formation is more prevalent. (Inset) A passing motion is used to define the characteristic velocity *V*_c_. In time Δ*t*, the trailing rider moves *w*_b_ and *kL*_b_ in the transverse and longitudinal directions, respectively. (Online version in colour.)

The wave propagation behaviour can be generalized by considering transverse and longitudinal wave speeds $V_{\phi_{T}}$, $V_{\phi_{L}}$, respectively, as a function of the mean distance between nearest neighbours normalized by a bike length, $\bar{\Delta s}/L_{b}$ (where *L*_b_ = 1.7 m is a typical bike length). In figure 3*a*, the wave speeds are normalized by mean peloton speed *V*_p_, which retains the difference in transverse and longitudinal wave speeds arising from the different propagation time scales (i.e. consistent with figure 2*e*). We aim to derive characteristic scales of longitudinal and transverse velocity that rationalize the difference between these wave speeds.

Rather than normalizing by the peloton velocity, which would be expected to characterize the response of a cyclist to a stimulus in the world frame, we consider a characteristic motion in the moving peloton frame. The inset of figure 3*b* shows a fundamental motion between two cyclists, defined by a relative longitudinal speed Δ*v* and relative transverse speed defined as

3.1 $$V_{\mathrm{trans}}=\frac{w_{b}}{kL_{b}}\Delta v,$$

where *w*_b_ is the width of a cyclist and *k* is a parameter to be determined empirically. A scale for the velocity difference Δ*v* can be derived from the relative acceleration *a* of the faster cyclist giving

3.2 $$\Delta v=\sqrt{aL_{b}},$$

(see electronic supplementary material for more details). Several characteristic accelerations are candidates for *a*, including the maximal braking deceleration and maximal forward acceleration of a cyclist. However, here we find the longitudinal motions associated with the wave behaviour are best characterized by a non-braking deceleration due to aerodynamic drag and gravity given by

3.3 $$a\equiv a_{d}=\frac{F_{\mathrm{drag}}+F_{\mathrm{gravity}}}{m}=\frac{(1/2)\rho_{\mathrm{air}}V_{p}^{2}C_{D}A+mg\sin\alpha }{m},$$

where *g* is gravitational acceleration, *α* is the road slope, *ρ*_air_ is air density and *C*_D_, *A* and *m* are a cyclist’s drag coefficient, area and mass, respectively. Inserting equation (3.3) into equation (3.2) defines the relative longitudinal velocity Δ*v*, which is in turn used in equation (3.1) to define the transverse velocity *V*_trans_. Normalizing $V_{\phi_{L}}/\Delta v$ and $V_{\phi_{T}}/V_{\mathrm{trans}}$, with *k* = 0.41 computed empirically, provides good collapse of the transverse and longitudinal wave speeds for $\bar{\Delta s}/L_{b}<1$, as shown in figure 3*b*.

Thus, the velocity which best characterizes longitudinal waves is that associated with a rider’s non-braking deceleration due to drag and local road slope. This velocity is significantly smaller in magnitude than that associated with braking, which implies that the riders are acting with the combined goals of safety and energy conservation. The transverse velocity is indicative of one rider passing another, rather than a maximum possible transverse speed associated with a stable turning motion [37] (electronic supplementary material). This indicates that the basic motion that collapses the wave speeds in the peloton is that of one rider passing another with a relative velocity characterized by the non-braking deceleration.

### 3.2. The role of vision

**Figure 4.**
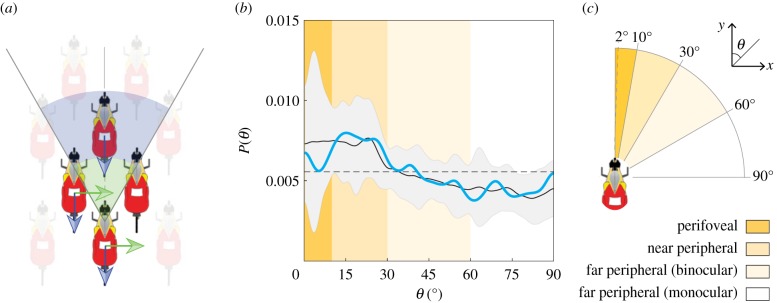
Information propagation and network structure are governed by the visual sensory system. (*a*) In the underlying diamond structure, the cyclist at the back reacts to transverse motions of nearest neighbours (green arrows), but reacts to longitudinal motions of the cyclist at the front of the diamond, two neighbours away (blue arrows). (*b*) Neighbouring cyclists have higher probability *P*(*θ*) of being oriented within *θ* ∈ [0, 30°]. The solid black curve is the mean probability from six different cases (electronic supplementary material, figure S2); light grey bands are 95% uncertainty bounds; light blue curve corresponds to figure 5*e*; horizontal dashed line is average *P*(*θ*) over all *θ*. (*c*) The range of the near-peripheral visual field corresponds to the most frequently occurring angles in the cyclist network (adapted from [39,43]; central 2° arc is foveal vision). (Online version in colour.)

We propose that the diamond-shaped lattice structure (seen in figure 1 and electronic supplementary material, figure S1) accommodates a mechanism of information transfer that results in the observed wave behaviour. Independent of long-term race goals, the persistent objectives of a cyclist are to stay in a beneficial drafting position (trivially satisfied inside the peloton [35]) and to avoid crashing. Crashes are most often caused by the sudden slowing of a rider located directly in front of another cyclist. The diamond structure separates the front-most cyclist, as shown in figure 4*a*, allowing the rider at the back of the diamond to effectively react to a backward propagating longitudinal wave two neighbours ahead, which is consistent with measured propagation times (figure 2*e*). Additionally, the nearest side-flanking neighbour is offset to the front providing more space for transverse motion as cyclists are not generally arranged shoulder-to-shoulder (electronic supplementary material, videos S3 and S4). We also note that if cyclists are responding to wave motions with a fixed reaction time Δ*t* = *t*_r_, then we would expect $V_{\phi }/V_{c}\propto \bar{\Delta s}/L_{b}$, where *V*_c_ is the characteristic velocity scale. That is, the wave speed is expected to increase linearly with spacing between riders $\bar{\Delta s}$, which is what we see for non end of race (non-EOR) conditions (as shown by blue, green and yellow data markers in figure 3). Thus, supported by measured wave propagation times and wave speeds, our interpretation of the diamond structure is consistent with cyclists responding to motion—of the nearest neighbour for transverse waves and two neighbours ahead for longitudinal waves—with a simple reaction time.

We suggest that this reaction time is consistent with preattentive visual processing. In preattentive vision, a large range of the human visual field is inspected in parallel without requiring a change in focal attention [38,39]. Although information processing capability is limited in preattentive visual processing, basic information along dimensions of texture, colour and motion can be handled in parallel and responded to rapidly [38–40]. Visual processing that requires focal attention over the limited range of the fovea occurs more slowly. Furthermore, changing focal attention requires as long as 200 ms if saccadic eye movements are required [38] (e.g. to change the gaze of the eye). In the context of cycling pelotons, transverse motion waves propagate from cyclist to cyclist in ≈250 ms, which necessarily encompasses the time taken to process a motion perturbation and to respond to it by moving. This time scale is consistent with simple human reaction time to visual stimuli [36]. Therefore, we conclude that cyclists are responding to motions of neighbours using preattentive visual processing without performing saccadic eye movements, which would result in longer wave propagation times than are observed.

This capability to respond to motions perceived outside the foveal field of vision (central field extending out to ≈± 2° [39]) is enabled by the ability of humans to detect motion in the near-peripheral field of view, with sensitivity to motion decreasing with increasing angular range (or eccentricity) [39,41,42]. Thus, if our interpretation of group structure is correct, we would expect cyclists to arrange themselves such that frontal neighbours in the diamond pattern are within a range of angles defined by horizontal peripheral vision, in order to perceive and accommodate motion perturbations. To test this, we measure the angle *θ* between each cyclist and their connected neighbours and do this for all cyclists on all frames within a given video clip (the analysis for each clip is summarized in electronic supplementary material, sections S2,S3 and figures S2–S6). The probability distributions measured over several peloton realizations show significantly higher likelihood that the angle takes a value *θ* ∈ [0, 30°], as shown in figure 4*b*. The drop in probability at *θ* ≈ 30° coincides with the limit of the human near-peripheral visual range (figure 4*c*) [39,43]. This angle is much larger than the maximal angle predicted for drafting benefit in a two-cyclist drafting situation (≈± 5°) [33]. Furthermore, the trend between the measured range of *θ* in pelotons and the range of the near-peripheral visual field holds even in slow, uphill riding scenarios where aerodynamic drag would be small due to the low speed, further suggesting that aerodynamics is not the main driver of intra-peloton structure (electronic supplementary material, figure S4). Further evidence for this proposed description of vision-based interaction is found by computing probability distributions of the angle between each sequential set of neighbours affected by propagating waves, which show similar roll-off for *θ*_wave_ > 30° (electronic supplementary material, figure S5). Lastly, we can compute a characteristic angle relative to the forward direction from the ratio of characteristic wave velocities, $\psi =\arctan(V_{\mathrm{trans}}/\Delta v)=\arctan(w_{b}/kL_{b})$, which gives *ψ* = 30.3° with the empirically found value of *k* = 0.41. This value is consistent with the bounds found in network structure measurements and lends further support for the role of near-peripheral vision in interaction.

**Figure 5.**
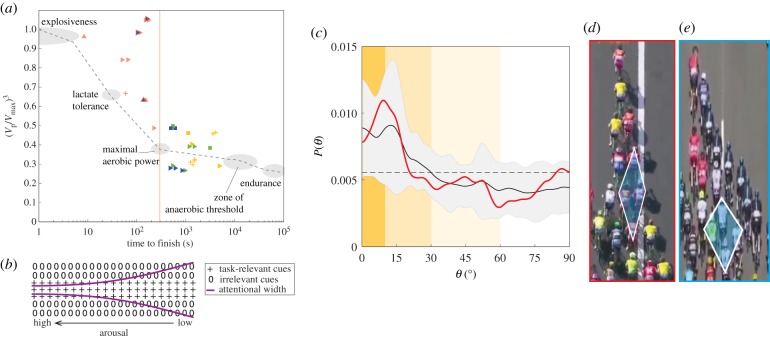
Physical exertion affects network structure and information propagation through arousal-dependent sensory function. (*a*) Normalized power output ((*V*_p_/*V*_max_)^3^; *V*_max_ is peak explosive speed) as a function of time to finish *t*_f_ (symbols same as for figure 3*a*; grey dashed curve and physical capacity zones adapted from [44]). (*b*) Easterbrook’s Cue Utilization Theory [45] predicts narrowing perception of task-relevant cues with increased arousal (e.g. via increased physical exertion). (*c*) For EOR conditions (*t*_f_ < 300 s), the roll-off in *P*(*θ*) occurs in a narrower range of the near-peripheral visual field (mean curves and uncertainty bounds are computed from seven cases shown in electronic supplementary material, figure S6). (*d*,*e*) Overhead images projected into a metric reference frame show a change in the diamond geometry (image credits: A.S.O. Eurosport, with permissions). (*d*) The peloton in EOR conditions has elongated diamond structures (image corresponds to red curve in (*c*)). (*e*). In non-EOR conditions, the diamond structures are wider and the echelon boundary angle is larger (image corresponds to blue curve in figure 4*b*). (Online version in colour.)

For nearly all variables, the wave speed is consistent with our description of information propagating with fixed inter-individual time scales such that $V_{\phi }/V_{c}\propto \bar{\Delta s}/L_{b}$. However, near the end of the race (EOR; time to finish *t*_f_ < 300 s), this trend breaks down and we find $V_{\phi_{T}}/V_{c}\approx \text{constant}$ for transverse waves, as shown by the transverse EOR (pink) data markers in figure 3*a*,*b*. This leads us to question if there is something fundamentally different in the sensory mechanisms affecting interaction principles during these conditions. We gain some insight by observing that the peak sustained power output over a duration of effort of 300 s coincides with cyclists entering into the maximal aerobic power zone of physical capacity [44], as shown in figure 5*a*. Following Easterbrook’s Cue Utilization Theory [45], several studies in sports psychology have linked increased arousal (through increased physical exertion) to a narrowing of individual perception of relevant task cues [46], figure 5*b* (see also electronic supplementary material). We suggest that the increasing power output associated with EOR conditions reduces the range of used sensory perception and predict that the internal group structure should narrow to reflect a reduction in used field of view. Measuring *θ* for connected neighbours shows a roll-off in probability at a narrower angle (≈20°, figure 5*c*) compared to non-EOR conditions (≈30°, figure 4*b*), supporting this prediction. The narrowing structure is evident in overhead images of the peloton and generally manifests in a shallower angle at the boundary of the global formations (figure 5*d*,*e*). The precise reason for the insensitivity of transverse wave speed to rider spacing near the EOR is not clear but can be interpreted as cyclists responding to a virtual obstacle moving at a fraction of their speed (see electronic supplementary material, figure S8 and section 3.3 for more description). Whether and how this relates to changes in sensory system function under increased arousal warrants further study. We also note that the trend in the speed of longitudinal waves is not affected in EOR conditions, implying that the imperative to not crash into the cyclist immediately in front is the prevailing concern in longitudinal wave motion regardless of race conditions.

### 3.3. Continuum modelling of cycling pelotons

**Figure 6.**
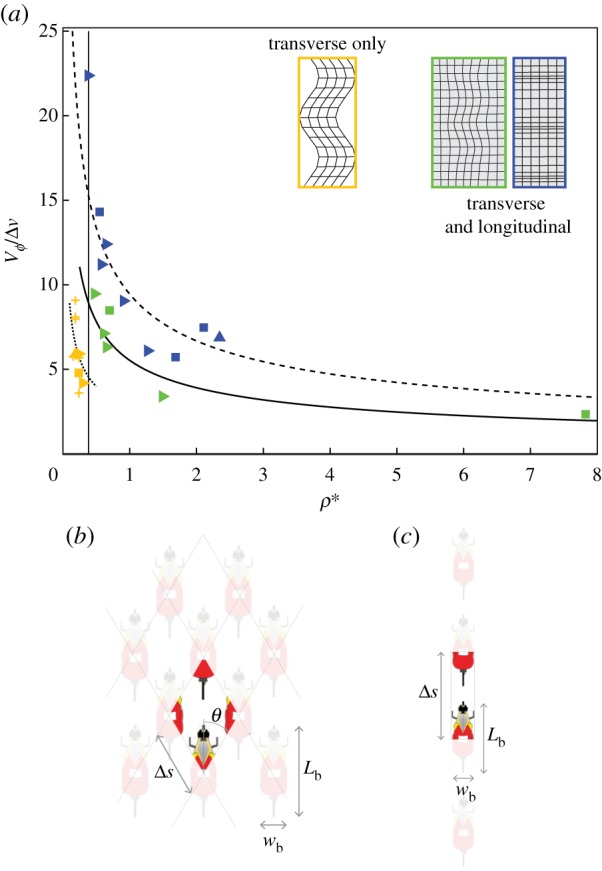
Wave behaviour can be described by analogous continuum models. (*a*) For $\bar{\Delta s}/L_{b}\leq 1$, a linear elastic solid model with E* = 75.8 and *σ* = 0.24 captures measured wave speeds as a function of ρ*. For $\bar{\Delta s}/L_{b}>1$ (data points left of the vertical line), a taut string model with T* = 7.9 captures the transverse wave speed. All symbol shapes are the same as in figure 3*a*. (*b*,*c*) Schematics showing how equivalent density ρ* is computed for application of the equivalent solid mechanics models for (b) a peloton spanning the road and (*c*) a line of cyclists. (*b*) Cyclists are shown in the diamond configuration with nominal angle *θ* defined as shown. The mean spacing between cyclists is $\bar{\Delta s}$. The density ρ* is defined for a unit diamond as the ratio of occupied area to an empty area. (*c*) For a line of cyclists, the same definition of the occupied area to open area is used for ρ*, where the centre to centre spacing between riders defines the unit cell. (Online version in colour.)

Although we have focused on how sensory mechanisms govern local interactions in pelotons, global models of collective groups are broadly useful in describing emergent behaviour. Indeed, collective behaviour often evokes analogous physical phenomena with researchers applying models motivated by thermodynamics [5], statistical mechanics [47] and vehicle traffic patterns [48] to describe various aspects of group dynamics. Here, we are motivated by other examples found in the natural world, wherein longitudinal and transverse waves propagate through a medium with different speeds, such as in seismology [49] and the behaviour of elastic materials [50]. In cycling pelotons, the fact that longitudinal and transverse waves have different speeds in the same group for $\bar{\Delta s}/L_{b}<1$ (figure 3) here motivates the application of a linear elastic solid model. For a linear elastic solid, transverse and longitudinal wave speeds are defined as $V_{\phi_{T}}=\sqrt{\left(E/2\rho (1+\sigma )\right)}$ and $V_{\phi_{L}}=\sqrt{\left(E(1+\sigma )/\rho (1+\sigma )(1-2\sigma )\right)}$, respectively, where *E* is the elastic modulus, *ρ* is the material density and *σ* is the Poisson’s ratio. To generate an analogous model for the cycling pelotons, we define an effective, dimensionless density ρ* as the ratio of the area occupied by riders to the open area in a two-dimensional plane projected onto the road, as shown in figure 6*b*. For a peloton with riders configured in the diamond pattern with nominal angular orientation defined by *θ*, the equivalent density can be derived as

3.4 $$\rho^{*}=\frac{L_{b}w_{b}}{{\bar{\Delta s}}^{2}\sin2\theta -L_{b}w_{b}},$$

where $\bar{\Delta s}$ is the average centre-to-centre distance between successive wave-affected cyclists, and a nominal value of *θ* = 30° is used to compute ρ* for all cases. Normalized transverse and longitudinal wave speeds are defined as

3.5 $$V_{\phi_{T}}^{*}\equiv \frac{V_{\phi_{T}}}{\Delta v}=\sqrt{\frac{E^{*}}{2\rho^{*}(1+\sigma )}}$$

and

3.6 $$V_{\phi_{L}}^{*}\equiv \frac{V_{\phi_{L}}}{\Delta v}=\sqrt{\frac{E^{*}(1-\sigma )}{\rho^{*}(1+\sigma )(1-2\sigma )}},$$

respectively, with analogous elastic modulus E*, density ρ* and Poisson’s ratio *σ*. Dividing equation (3.6) by equation (3.5) gives

3.7 $$\sigma =\frac{{\left((V_{\phi_{L}}/V_{\phi_{T}})\right)}^{2}-2}{2{\left((V_{\phi_{L}}/V_{\phi_{T}})\right)}^{2}-2}.$$

The ratio of characteristic longitudinal to transverse wave speed Δ*v*/*V*_trans_ = *kL*_b_/*w*_b_ (with *k* = 0.41) can be substituted into equation (3.7) for $V_{\phi_{L}}/V_{\phi_{T}}$ to estimate *σ*, which gives 0.24. A nonlinear least-squares fit to equations (3.5) and (3.6) using measured longitudinal and transverse wave speeds at different observed values of ρ* can then be used to estimate the analogous elastic modulus, which gives E* = 75.8. The resulting model fits to $V_{\phi_{T}}^{*}$ and $V_{\phi_{L}}^{*}$ are shown in figure 6*a*, which capture the measured wave speed data.

For $\bar{\Delta s}/L_{b}>1$, cyclists tend to ride in a line and no longitudinal waves exist. In this case, a taut string model is more appropriate, for which the normalized wave speed is defined as

3.8 $$V_{\phi_{T}}^{*}=\frac{V_{\phi_{T}}}{\Delta v}=\sqrt{\frac{T^{*}}{\rho^{*}}},$$

where T* is a normalized tension. Here, the dimensionless density is again defined as the ratio of occupied area to open area projected into the road plane, but now with riders in a line (figure 6*c*). The density is thus defined as

3.9 $$\rho^{*}=\frac{L_{b}w_{b}}{\bar{\Delta s}w_{b}-L_{b}w_{b}}=\frac{L_{b}}{\bar{\Delta s}-L_{b}}.$$

Performing a nonlinear least-squares fit to equation (3.8) gives T* = 7.9 and results in the fit shown in figure 6*a*. Thus, linear elastic solid mechanics models can be reasonably applied to describe the wave propagation behaviour in cycling pelotons. One caveat to point out is that while an elastic solid model allows for backward and forward wave propagation, we do not observe forward propagating waves in cycling pelotons. This is presumably due to the fact that interactions between anterior and posterior cyclists are non-reciprocal, as is also the case in other collective groups (e.g. [4,48]). Nonetheless, the elastic solid model applied here relates discernible properties of intra-peloton structure and agent spacing to observations of wave-like motions within the peloton.

## 4. Conclusion

Our findings show how interaction principles in dense cycling pelotons are governed by the human visual sensory system. The angular range of near-peripheral vision, which is sensitive to motion, sets the internal diamond lattice structure that pervades pelotons. This structure safely accommodates motion perturbations that result in transverse and longitudinal waves whose speed can be described by a linear elastic solid model. The diamond pattern supports longitudinal waves that propagate at twice the speed of transverse waves as cyclists respond to longitudinal motions of the cyclist at the forward point of the diamond (two neighbours away), while responding to transverse motions of their nearest side-flanking neighbour. Near the end of the race (EOR), the wave propagation behaviour changes and the internal structure narrows. This effect appears to be the result of a narrowing of sensory focus associated with higher energetic output.

Scientific interest in natural collective behaviour has been high for some time, but a robust understanding of the interaction principles between agents has been lacking. As autonomous engineered capabilities continue their rapid ascent, questions of how best to define interactions between autonomous agents rise to the forefront. The interaction principles revealed in cycling pelotons connect sensory systems to emergent collective behaviour, suggesting that the internal group structure is an emergent effect of sensory properties. This promises to be a useful framework for describing how, for example, a swimming fish school rapidly transitions to evasive behaviour, or how a collection of self-driving cars or autonomous robots can be programmed to adapt to evolving environments.

## Supplementary Material

Supplementary text and figures for How vision governs the collective behavior of dense cycling pelotons

## Supplementary Material

Supplementary Video S1 for How vision governs the collective behavior of dense cycling pelotons

## Supplementary Material

Supplementary Video S2 for How vision governs the collective behavior of dense cycling pelotons

## Supplementary Material

Supplementary Video S3 for How vision governs the collective behavior of dense cycling pelotons

## Supplementary Material

Supplementary Video S4 for How vision governs the collective behavior of dense cycling pelotons

## Data accessibility

The datasets generated and analysed during the current study are publicly available and are hosted by Utah State University on their Digital Commons: https://digitalcommons.usu.edu/all_datasets/70/.
