# Supplementary material for: How vision governs the collective behaviour of dense cycling pelotons
Source: J R Soc Interface. 2019 Jul 10;16(156):20190197. doi: 10.1098/rsif.2019.0197 (PMC6685023; doi:10.1098/rsif.2019.0197)
Supplement: Supplementary text and figures for How vision governs the collective behavior of dense cycling pelotons [file rsif20190197supp1.pdf]

How vision governs the collective behavior of dense cycling pelotons: Supplementary Material

J. Belden[1], M. M., Mansoor[2], A. Hellum[1], S. R., Rahman[2], A. Meyer[3], C. Pease[4], J. Pacheco[5], S. Koziol[6] & T. T., Truscott[2]

[1]Naval Undersea Warfare Center, Newport, RI 02841
[2]Dept. of Mechanical and Aerospace Engineering, Utah State University, Logan, UT 84322
[3]Robbins College of Health and Human Sciences, Baylor University, Waco, TX 76798
[4]VeloCam Services
[5]CSAIL, Massachusetts Institute of Technology, Boston, MA 02139
[6]School of Engineering and Computer Science, Baylor University, Waco, TX 76798

*To whom correspondence should be addressed; E-mail: jessebelden@gmail.com.

Table S1. List of symbols used in the main text.

Symbol	Definition
A	cyclist's projected area
a	relative acceleration between two cyclists
α	road slope
C_D	cyclist's drag coefficient
Δs	center-to-center distance between neighboring cyclists
$\overline{\Delta s}$	mean distance between neighboring wave-affected cyclists
Δv	relative longitudinal speed between two cyclists
E	elastic modulus
E^*	analogous elastic modulus in cycling pelotons
g	gravitational acceleration
k	empirical parameter relating characteristic velocity scales
L_b	bike length
m	cyclist's mass
N	number of cyclists affected by wave
ψ	characteristic angle associated with wave propagation
$P(\theta)$	probability distribution of θ
ρ	material density
ρ^*	analogous density in cycling pelotons
ρ_{air}	air density
σ	Poisson's ratio
T^*	analogous tension in cycling pelotons
t	time
t_f	time to finish
t_r	human simple visual reaction time
t_w	wave propagation time
θ	angle between neighboring cyclists
θ_{wave}	angle between each sequential set of neighbors affected by a propagating wave
V_c	characteristic wave speed
V_{max}	peak explosive speed of a cyclist
V_p	mean peloton velocity
V_ϕ	wave speed
V_{ϕ_L}	longitudinal wave speed
$V^*_{\phi_L}$	normalized longitudinal wave speed
V_{ϕ_T}	transverse wave speed
$V^*_{\phi_T}$	normalized transverse wave speed
V_{trans}	relative transverse speed between two cyclists
w_b	cyclist width

1 Global formations of cycling pelotons

Figure S1 shows different prevailing shapes that the peloton takes on in the Tour de France (TdF) professional cycling stage race.

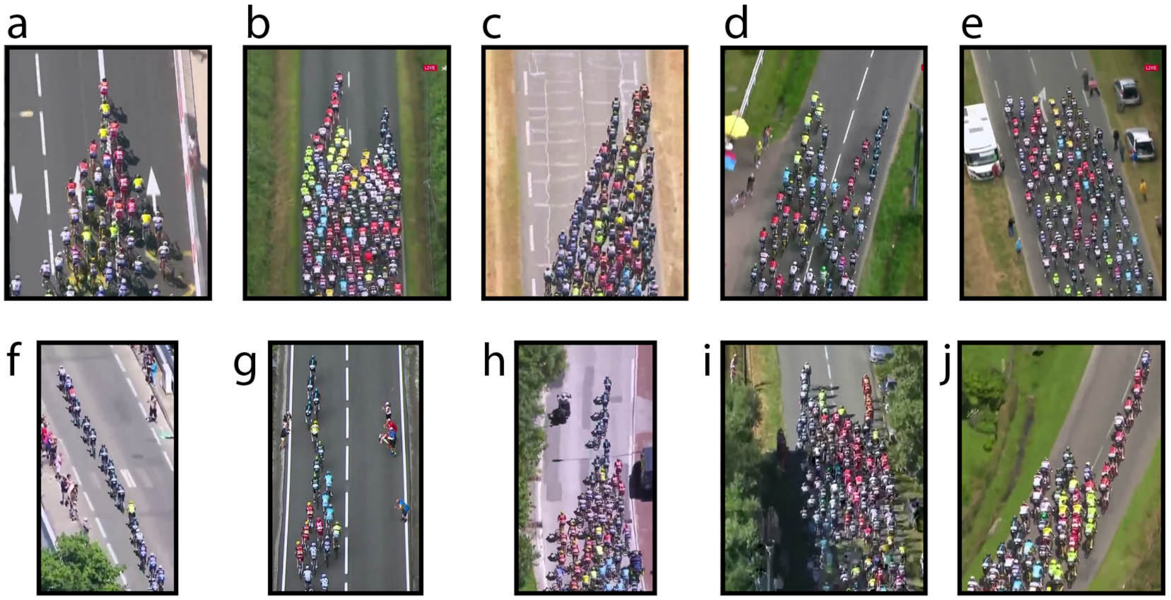

Figure S1. Different persistent formations in Tour de France pelotons are described by the boundary head shape at the front: **a,** arrow, **b,** double arrow, **c,** echelon, **d,** double echelon, **e,** flat, **f,** line, **g,** line into arrow, **h,** line into echelon, **i,** double line into echelon, **j,** line into flat. Image credits: A.S.O. Eurosport, with permissions.

2 Image Processing

Cyclist position data are resolved from video sequences using a series of image processing algorithms shown in Figure S2. Video sequences are first parsed into individual images and bounding boxes for each rider are defined by a user for the first frame in an image sequence. A tracking routine built on the track-learn-detect (TLD) algorithm [1] then tracks each initialized rider through the sequence. All data are manually post-processed to fix any errant tracks or

missed riders, and then the position of each cyclist is computed in pixel coordinates as the centroid of the bounding box. In order to make measurements of relative positions or angles, the coordinates need to be transformed into a metric reference frame. We accomplish this by generating homographies [2] between the raw images and reference geometry, which herein is established using known lengths and distances of lane markings on the road (Figure S2(c)). These homographies can then be used to transform pixel coordinates in the image reference frame to physical coordinates in a metric (world) reference frame as $\mathbf{x_c} = H\mathbf{x_{pix}}$, where H is a 3×3 homography matrix, and $\mathbf{x_c}$ and $\mathbf{x_{pix}}$ are cyclist centroid coordinates in the world and image reference frames, respectively. Following the transformation, the y axis is aligned with the longitudinal road direction and the x axis is aligned transverse to the road direction.

To measure the angle between neighboring riders, a Delauney triangulation is performed on all transormed cyclist coordinates ($\mathbf{x_c}$) to form a connected network between all cyclists and their neighbors (Figure S2(d)). Angles are defined with respect to the y axis (forward road direction) and are reported as absolute values such that $\theta \in [0°, 180°]$. Angles between $90° < \theta \leq 180°$ correspond to riders outside a cyclist's field of vision and thus we only plot $0° \leq \theta \leq 90°$ in figures 4 & 5 of the main text and figures S3, S4, S5 & S6. The angles are only computed for connected riders that are within $2L_b$ of one another so as to exclude extreme angles that can occur between boundary nodes in the connected network (Figure S2(d)). These measurements are made for video sequences at several different points in the race with different realizations of the global peloton formation. For each sequence, angle measurements are made for $N_{cyclists}$ number of cyclists and N_t number of time instances, with spacing between instances of $\frac{1}{30}$ sec. From these data, the probability distribution function $P(\theta)$ is computed by fitting a smoothing spline to the discrete cumulative distribution function CDF, and then taking the derivative to get a best fit estimate of $P(\theta)$. The values of $P(\theta)$ for all cases studied herein are plotted in figures S3, S4 & S6 and the values of $N_{cyclists}$ and N_t are reported in the caption for

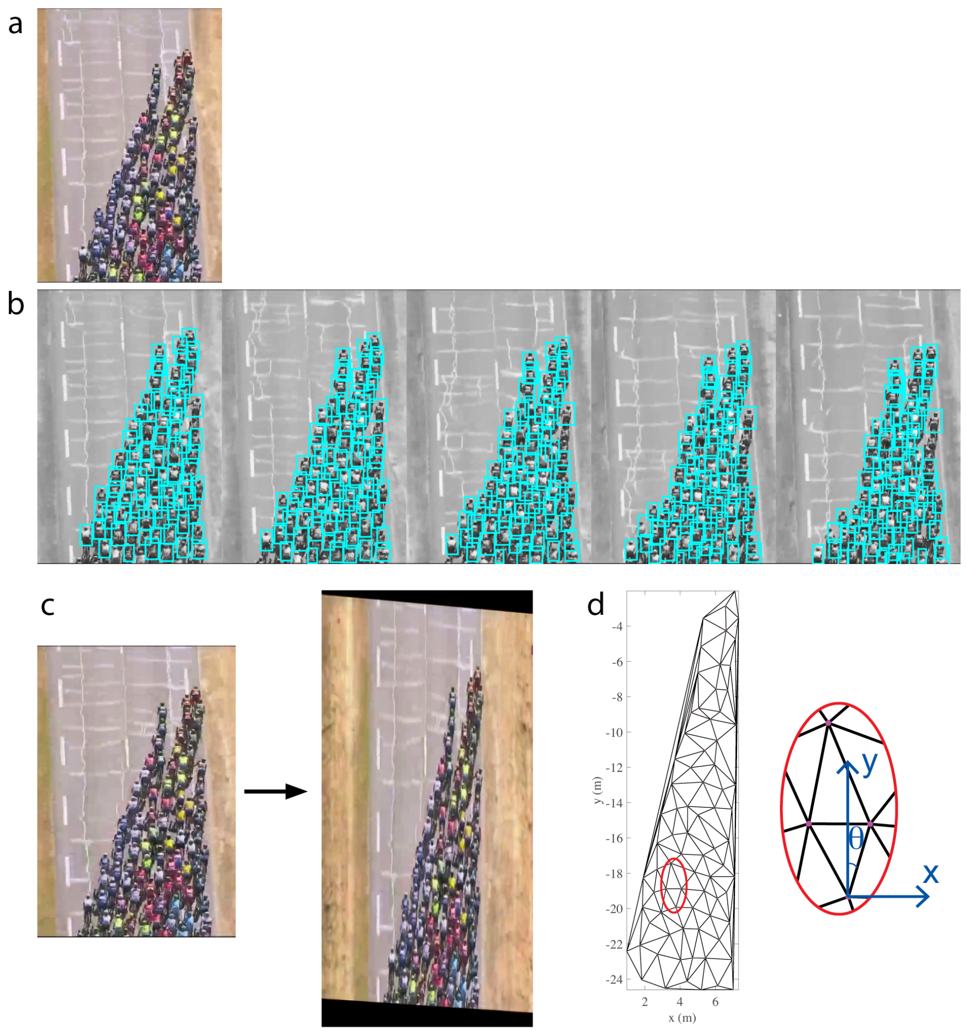

Figure S2. Image processing algorithms were used to track the cyclists in each frame and transform tracked coordinates to a metric reference frame. **a,** An original image from the helicoptor video clip. **b,** Sample results of the tracking algorithm used to detect cyclists' locations through time in a given video sequence. The left most image corresponds to **(a)**, and the image sequence is the same as shown in figure 2(a) in the main text. Initial tracks were automatically determined with an algorithm built on that of Kalal et al. [1], with corrections made manually thereafter. A bounding box (shown in cyan) is drawn around each cyclist with the centroid of the bounding box taken as the rider's coordinates. **c,** Images from the overhead view are projected into a metric reference frame using the geometry of road lane markings as reference geometry. **d,** Delauney triangulation is used to determine the connectivity of neighbors in the peloton. The angles between each cyclist and their connected neighbors are computed and used to determine $P(\theta)$, $CDF(\theta_{wave})$ and $P(\theta_{wave})$. Note that the angles are only computed for connected riders that are within $2L_b$ of one another so as to exclude extreme angles that can occur between boundary nodes in the connected network. Image credits: A.S.O. Eurosport, with permissions.

each case. Figure 4(b) in the main text plots the mean of all $P(\theta)$ curves for all non-end of race (non-EOR) echelon/arrow head cases shown in figure S3. The uncertainty bounds reported in figure 4(b) are computed by propagating uncertainty as

$$u_{\text{total}} = \pm\sqrt{\sum_{j=1}^{M} u_{P_j}^2 + \left(t_{M-1,95\%} S_{P_c}\left(\theta\right)\right)^2} \tag{1}$$

where $u_{P_j} = t_{\nu,95\%} S_{yx}$, with S_{yx} the standard error of the spline fit of $P(\theta)$ to the discrete probability distributions for each case and $t_{\nu,95\%}$ is the value from the student's t-distribution table for ν degrees of freedom and 95% confidence; here $\nu = N - (m+1)$, where N is the number of discrete points and m is the order of the spline fit. In the second uncertainty term in Eq.1, $S_{P_c}\left(\theta\right)$ is the standard deviation of $P(\theta)$ for each case from the mean value computed for all cases and M is the number of cases. Figure 5(c) in the main text plots the mean of all $P(\theta)$ curves for all EOR cases shown in figure S6 with the uncertainty bounds computed in the same way.

3 Wave propagation

Supplementary Video 1 shows a transverse wave that is initiated at the boundary of the peloton and propagates through the group. This video corresponds to the case shown in figure 2(a) of the main text and figure S2(b). Video credit: A.S.O. Eurosport, with permissions.

Supplementary Video 2 shows several longitudinal waves propagating within the peloton. The waves are initiated by motions of cyclists avoiding a backward moving rider. The video plays again at half speed and highlights two longitudinal waves. This video corresponds to the case shown in figure 2(c) of the main text. Video credit: A.S.O. Eurosport, with permissions.

Supplementary Video 3 shows a view from a rear-facing GoPro camera mounted to a rider's

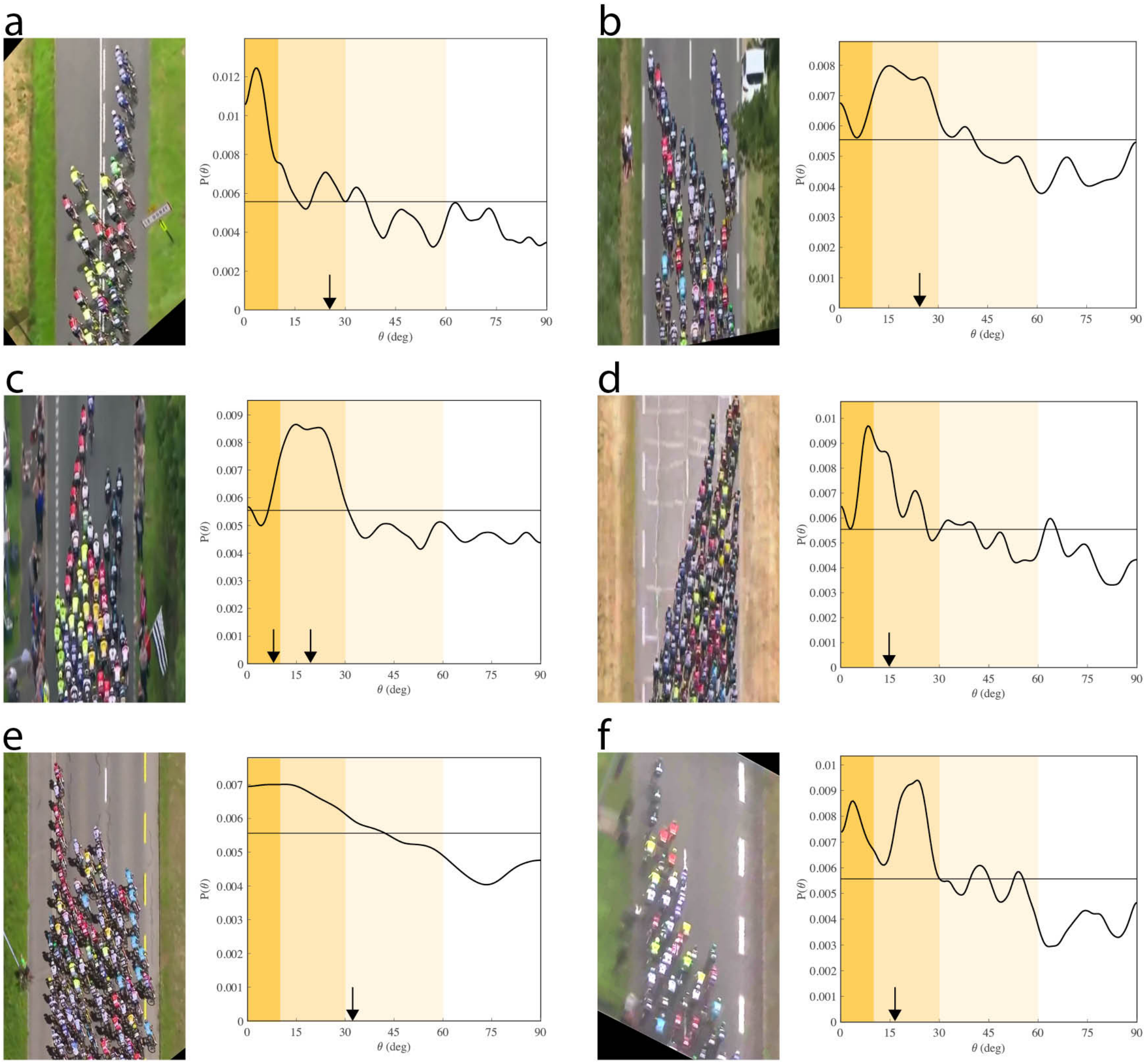

Figure S3. Probability distributions of angular location of neighbors $P(\theta)$ for cases shown in figure 4(b) of the main text. These cases were used to compute the mean and uncertainty on $P(\theta)$ shown in figure 4(b); each case has measurements for number of cyclists $N_{cyclists}$ and number of time instances N_t, with spacing between instances of $\frac{1}{30}$ sec. The horizontal black line in each plot denotes the mean value of $P(\theta)$ over all θ. The black arrows denote the front peloton boundary angles with respect to the forward direction for echelons (1 arrow) or arrow head (2 arrows). Head shape, number of cyclists and number of time steps for each case are: **a,** echelon, $N_{cyclists} = 31$, $N_t = 179$; **b,** echelon, $N_{cyclists} = 45$, $N_t = 343$; **c,** arrow, $N_{cyclists} = 63$, $N_t = 189$; **d,** echelon, $N_{cyclists} = 118$, $N_t = 183$; **e,** echelon, $N_{cyclists} = 106$, $N_t = 125$; **f,** echelon, $N_{cyclists} = 45$, $N_t = 147$. All overhead images have been projected into a metric reference frame. Image credits: A.S.O. Eurosport, with permissions.

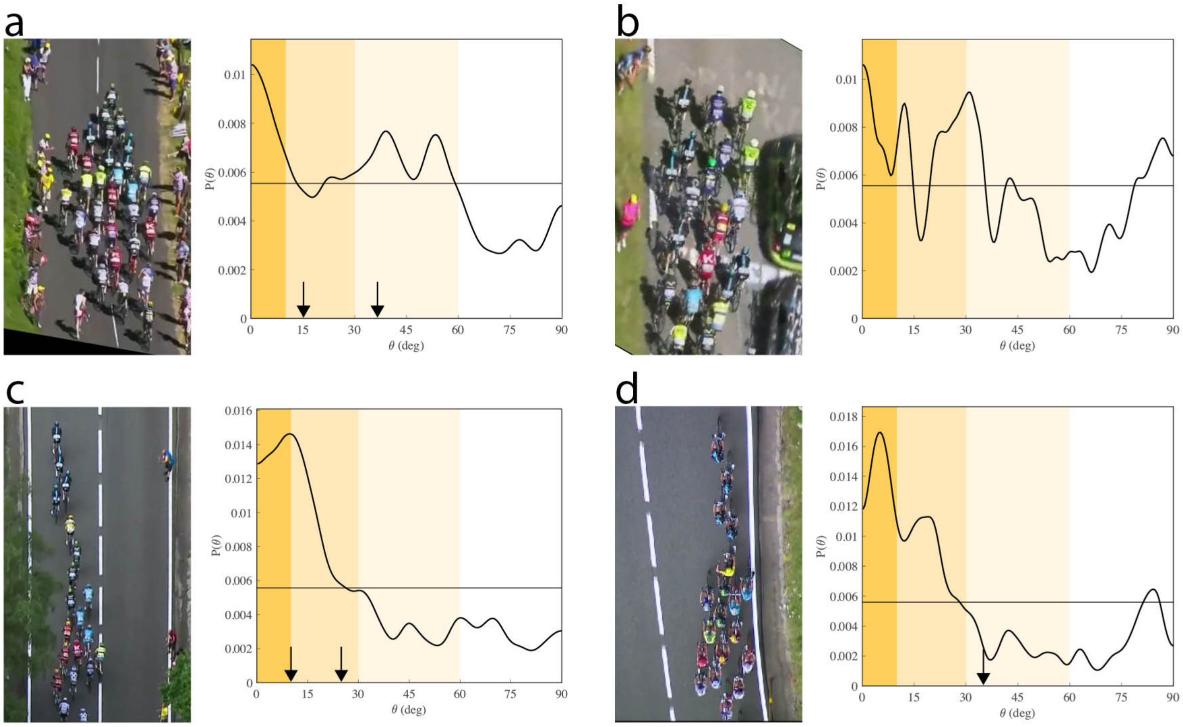

Figure S4. Probability distribution of angular location of neighbors $P(\theta)$ for 4 uphill-riding peloton cases. The horizontal black lines and black arrows denote the same things as in figure S3; the boundary angle for the flat head case is not marked. Head shape, road slope, number of cyclists and number of time steps for each case are: **a,** arrow, $\alpha = 7.5°$, $N_{cyclists} = 30$, $N_t = 218$; **b,** flat, $\alpha = 9.5°$, $N_{cyclists} = 18$, $N_t = 298$; **c,** line into arrow, $\alpha = 5.5°$, $N_{cyclists} = 27$, $N_t = 152$; **d,** line into arrow, $\alpha = 6.5°$, $N_{cyclists} = 18$, $N_t = 194$. All overhead images have been projected into a metric reference frame. Image credits: A.S.O. Eurosport, with permissions.

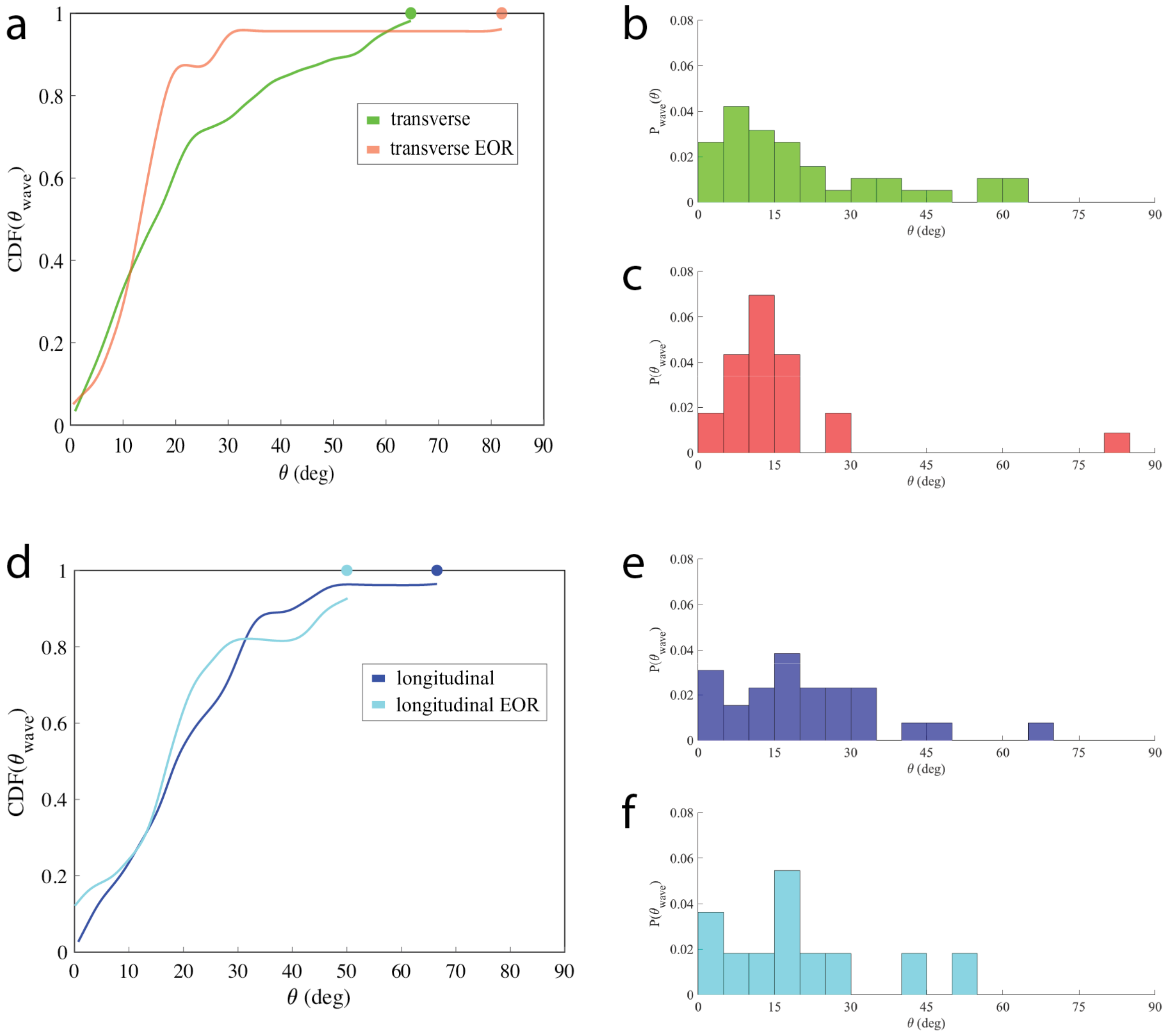

Figure S5. Cumulative distribution functions CDF and histograms of relative angular orientation of wave-affected riders. The angle between successive neighbors affected by a propagating wave is denoted by θ_{wave}, which is defined with respect to the forward road direction. **a,** CDF of θ_{wave} for transverse waves in non end of race (non-EOR) and EOR conditions; curves are spline fits to the discrete CDFs and circle data markers show the last data point from the discrete CDFs. Discrete histograms showing relative frequency of occurrence of θ_{wave} are given for **b** non-EOR and **c** EOR conditions. The range of most likely angles narrows significantly in EOR conditions. The same plots are shown in **d,e,f** for angular orientation of riders affected by longitudinal waves. The trends between EOR and non-EOR are the same, but the effect not as pronounced as shown by the transverse waves. The number of wave-affected cyclists for each case analyzed are: **b,** transverse non-EOR, $N_{cyclists} = [7, 7, 5, 10, 4, 3, 4, 4, 3]$ corresponding to 9 cases analyzed; **c,** transverse EOR, $N_{cyclists} = [7, 5, 3, 5, 3, 4, 3]$; **e,** longitudinal, $N_{cyclists} = [7, 5, 3, 6, 5, 6]$; **f,** longitudinal EOR, $N_{cyclists} = [7, 4, 3]$.

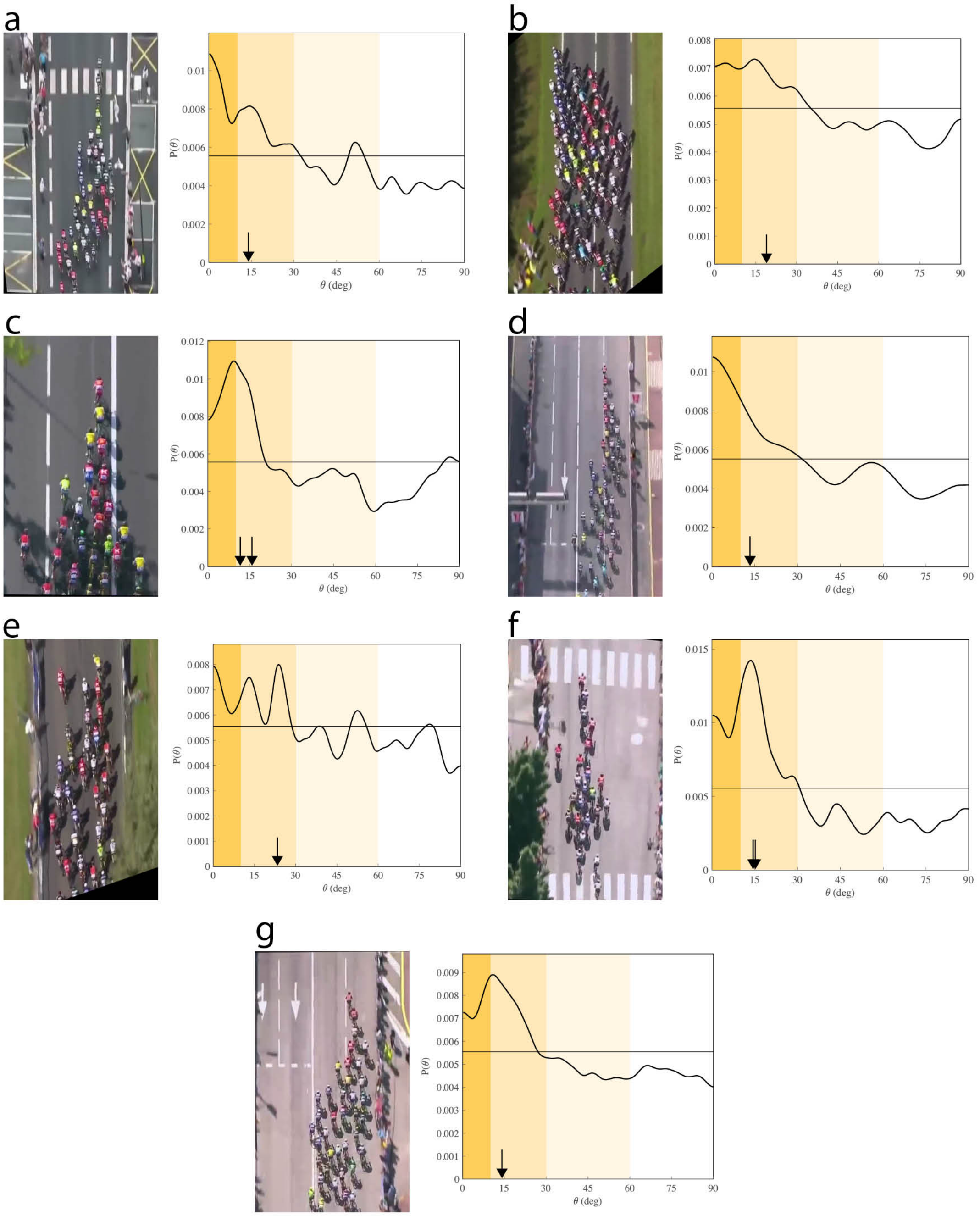

Figure S6. Probability distribution of angular location of neighbors $P(\theta)$ for EOR cases shown in figure 5(c) of the main text. These cases were used to compute the mean and uncertainty on $P(\theta)$ shown in figure 5(c). The horizontal black lines and black arrows denote the same things as in figure S3. Head shape, number of cyclists and number of time steps for each case are: **a,** echelon, $N_{cyclists} = 44$, $N_t = 250$; **b,** echelon, $N_{cyclists} = 73$, $N_t = 101$; **c,** arrow, $N_{cyclists} = 20$, $N_t = 166$; **d,** echelon, $N_{cyclists} = 50$, $N_t = 157$; **e,** echelon, $N_{cyclists} = 32$, $N_t = 193$; **f,** line into arrow, $N_{cyclists} = 21$, $N_t = 239$; **g,** echelon, $N_{cyclists} = 38$, $N_t = 150$. All overhead images have been projected into a metric reference frame. Image credits: A.S.O. Eurosport, with permissions.

bicycle shows the basic diamond structure. In this clip, cyclists brake hard in response to a stimulus up the road. The diamond configuration helps avoid a catastrophic crash. Video credit: GoPro World.

Supplementary Video 4 shows a view from a forward-facing GoPro camera mounted to a rider's handlebars shows the diamond pattern and accommodation of a transverse motion. The cyclist to the left front flank moves to the right, prompting a transverse motion of the cyclist carrying the GoPro. Video credit: GoPro World.

3.1 Measuring wave properties

Longitudinal and transverse waves are identified visually from image sequences of helicopter TdF footage. Wave-affected cyclists are first identified on images projected into a metric reference frame (figure S7(a)). Then, the position of each rider relative to the instantaneous centroid of all riders is plotted for each frame in the sequence, as shown in figure S7(b). These data combined with visual inspection are used to determine the frame at which each affected rider first moves in response to the wave. The displacement of the wave front relative to the instantaneous location of the first wave-affected rider is plotted against time and fit with a line to determine the wave speed, as shown in figure S7(c). In addition to measuring the wave speed, the center-to-center distance between successive wave-affected cyclists Δs is measured on the frame on which the wave is initiated. The mean value of Δs between successive cyclists affected by the wave is computed on this frame and reported as $\overline{\Delta s}$ in figure 3 of the main text.

Finally, θ_{wave} is computed as the angle between a wave-affected cyclist and his nearest wave-affected neighbor to the front. Different instances of observed wave events are grouped into transverse non-EOR, transverse EOR, longitudinal non-EOR and longitudinal EOR. Figure

S5 plots smoothing spline fits to the discrete CDF of θ_{wave}, as well as the discrete probability distributions, showing that the range of angles between wave-affected riders narrows in EOR conditions.

3.2 Defining characteristic wave propagation velocities

We aim to derive characteristic scales of longitudinal and transverse velocity that rationalize the difference between these wave speeds shown in figure 3(a) of the main text. Consider two cyclists within a pack traveling with mean group velocity V_p. A velocity difference Δv in the longitudinal direction exists between the cyclists. Each cyclist has length and width L_b and w_b, respectively. A scale for the velocity difference Δv can be derived from the relative acceleration a of the faster cyclist, as follows.

$$\Delta v = at_p \tag{2}$$

with

$$t_p = \frac{L_b}{\Delta v} \tag{3}$$

Because L_b is used for the length scale in Eq.3, the time scale t_p may be interpreted as the time required for the faster cyclist to draw even with the slower one. We now have

$$\Delta v = \sqrt{aL_b} \tag{4}$$

such that Δv is the velocity scale characteristic of longitudinal wave-like motions within the pack. This is not the only choice of longitudinal velocity scale; two other obvious ones being $V_{c_L} = V_p$ and $V_{c_L} = a(L_b/V_p)$. However, these alternative scales include the peloton velocity V_p, which would be expected to characterize the response of a cyclist to a stimulus in the world frame rather than the moving peloton frame.

Physical arguments can be used to obtain candidates for the characteristic acceleration used in Eq.4. A maximal braking deceleration $a_b = 0.56g$ was calculated by Wilson et al. [3] for a

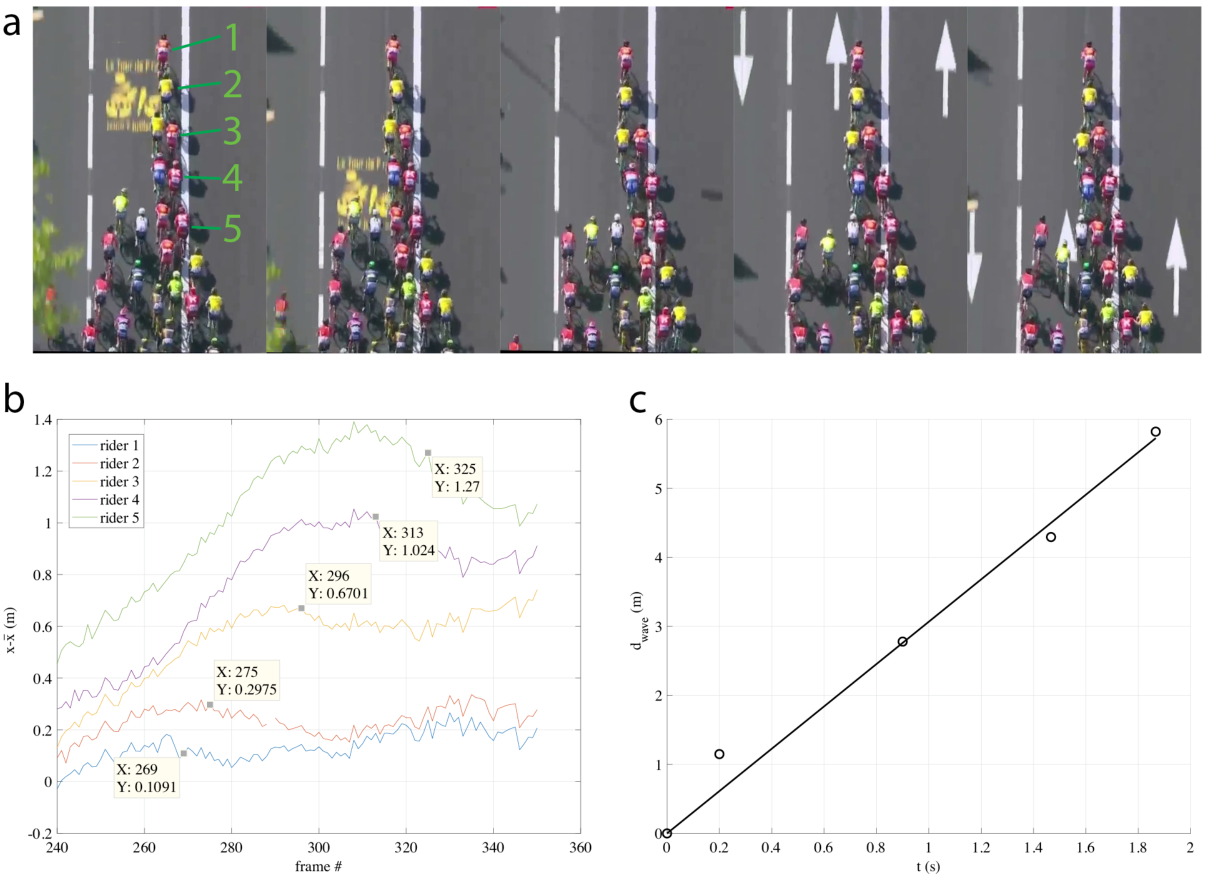

Figure S7. Processing routine used to measure wave propagation time and wave speed. **a,** Wave-affected cyclists are identified through visual inspection of a video sequence. All overhead images have been projected into a metric reference frame (image credits: A.S.O. Eurosport, with permissions). **b,** The position of each cyclist x relative to the centroid of the group $\bar{x}$ is plotted for each frame in the sequence and is used to help identify the location of the wave front. **c,** The distance of the wave front relative to the instantaneous location of the first affected cyclist is plotted against time and fit with a line to determine the wave speed.

nominal cyclist, where g is gravitational acceleration. A maximal non-braking deceleration a_d can be determined from the forces acting to slow the cyclist,

$$a_d = \frac{F_{\text{drag}} + F_{\text{gravity}}}{m} = \frac{\frac{1}{2}\rho_{\text{air}} V_p^2 C_D A + mg \sin\alpha}{m} \tag{5}$$

where m, C_D and A are the cyclist's mass, drag coefficient, and characteristic area. The local fluid density and road slope are given by ρ_{air} and α, respectively. Herein, the product $C_D A = 0.32$, as reported by Wilson et al. [3] for an individual cyclist. The total decelerating force $F_d = F_{\text{drag}} + F_{\text{gravity}}$ also determines a maximal forward acceleration

$$a_f = \frac{\dot{W}_{\max} - F_d V_p}{m V_p} \tag{6}$$

where $\dot{W}_{\max}$ is the maximum power output of the cyclist, estimable on a per-mass basis [3]. Each candidate acceleration has a different relationship to the velocity V_p

$$\begin{aligned} \frac{da_b}{dV_p} &= 0, \\ \frac{da_d}{dV_p} &> 0, \\ \frac{da_f}{dV_p} &< 0 \end{aligned} \tag{7}$$

The longitudinal motions of the cyclists studied in this work were deemed to be best characterized by a_d. This is interesting, because for typical racing speeds $a_b > 2a_d$, meaning that the wave speeds displayed by the cyclists in the longitudinal direction are not dictated by braking deceleration, which gives the largest velocity scale. Rather, the characteristic scale is consistent with energy preservation.

A geometric argument can be used to characterize the transverse motion. Consider the characteristic passing motion shown in figure 3(b) of the main text. For two cyclists to pass without penetration, a transverse motion equal to or greater than their width w_b is required. Over the time Δt, the "pass" is characterized by a transverse motion of the trailing rider of w_b, and a

longitudinal motion of kL_b, in the pack-fixed frame, where k is a parameter to be determined. The transverse velocity associated with this motion is $V_{trans} = w_b/\Delta t$ and the longitudinal velocity is $\Delta v = kL_b/\Delta t$. Eliminating Δt, these velocity scales can thus be related as

$$V_{trans} = \frac{w_b}{kL_b}\Delta v \tag{8}$$

This indicates a linear relationship between the transverse and longitudinal velocity scales. The value of the constant k that provides the best data collapse in figure 3(b) was empirically found to be $k = 0.41$. A characteristic angle can be computed from the ratio of characteristic wave velocities, $\psi = \arctan(V_{trans}/\Delta v) = \arctan(w_b/kL_b)$, which, when inserting $k = 0.41$, gives $\psi = 30.3°$; this value is consistent with the bounds found in network structure measurements.

The speed of these transverse and longitudinal motions that are found to characterize wave propagation are considerably slower than an estimate of the physical limit on velocities would indicate. For longitudinal motions, the physical limit would be set by braking deceleration, which, as noted above, was not found to characterize the observed wave propagation. In the trasverse direction, one might expect velocities to be limited by stability in turning. Performing an extrapolation of the stability analysis in Meijaard et al. [4] gives a velocity that is much too large to characterize transverse wave propagation. This indicates that the trajectory associated with one rider passing another is more characteristic of the wave speeds within the peloton than the individual velocities associated with maximum longitudinal and transverse motion.

3.3 Interpretations of wave behavior in cycling pelotons

To rationalize the expected dependence of wave speed V_ϕ on rider spacing, consider two cyclists separated by a distance of Δs. The trajectory of the first cyclist is $(s(t), n(t))$, where s and n refer to the longitudinal and transverse directions, respectively. For the case when a perfect wave-like motion is observed, the trajectory of the second cyclist is $(s(t + \Delta t_s), n(t + \Delta t_n))$.

For small changes in position,

$$V_{\phi_L} = \frac{\Delta s}{\Delta t_s}, \quad (9)$$

$$V_{\phi_T} = \frac{\Delta s}{\Delta t_n}, \quad (10)$$

where V_{ϕ_L} and V_{ϕ_T} are the wave phase velocities in the longitudinal and transverse directions, respectively. Each Δt represents the time it takes for the second cyclist to repeat the motions of the first. We refer to a general phase velocity V_ϕ and a general Δt with the understanding that we can associate these with a pattern of behavior in an arbitrary direction.

We can make the general forms of Eq.9 & 10 non-dimensional such that

$$\frac{V_\phi}{V_p} = \left(\frac{L_b}{V_p \Delta t}\right) \frac{\Delta s}{L_b}. \quad (11)$$

where V_p is the peloton velocity and L_b is the bike (or "body") length. A few manipulations of Eq.11 follow which have interesting interpretations for different observed wave propagation behavior.

Constant positive slope. For cyclists reacting to wave motion with fixed reaction time $\Delta t = t_r$, we can re-write Eq.11 as

$$\frac{V_\phi}{V_p} = \left(\frac{L_b}{V_p t_r}\right) \frac{\Delta s}{L_b}. \quad (12)$$

Thus, we can see that the wave speed is expected to increase linearly with spacing between riders Δs, which is what we see for non end of race (non-EOR) conditions (as shown by blue, green and yellow data markers in figure 3. The longitudinal waves propagate faster than the transverse waves because cyclists are responding to longitudinal perturbations 2 neighbors ahead (larger Δs), as discussed in the main text.

Zero slope. Consider a group of points where

$$\frac{V_\phi}{V_p} = \left(\frac{L_b}{V_p \Delta t}\right) \frac{\Delta s}{L_b} = \kappa \quad (13)$$

where κ is a constant. This is the trend shown by transverse waves in the end of race (EOR) conditions represented by the red data markers in figure 3. Manipulating Eq.13, we can relate the timescale of repeated motion to spacing as

$$\Delta t = \frac{\Delta s}{\kappa V_p}. \tag{14}$$

Thus, for the behavior observed in EOR conditions, the characteristic timescale associated with wave propagation increases linearly with spacing between riders. The special case $\kappa = 1$ is instructive. Here, $\Delta t = \Delta s / V_p$, such that the reaction time is equal to the time it takes for each body to cover the space separating them. This is analogous to the situation observed when a streamline encounters a fixed obstacle in a fluid flow. This can be generalized to the more general case $\kappa > 0$, as shown in figure S8. Here, it becomes clear that if a pattern of behavior is such that the cyclists have a fixed non-dimensional wave speed κ, it is equivalent to a response to a stimulus which is moving in their same direction with speed $(1 - \kappa)V_p$.

4 Cue Utilization Theory in the context of sports psychology

Sport psychology and performance-based examinations of the relationship between arousal (activation) states and field of perceptions (related to awareness of various cues) have been conducted to determine how arousal affects performance awareness. Easterbrook's Cue Utilization Theory [5] was the first to predict that as arousal increases, individual perception of relevant task cues narrows. This theory suggests, supported by many subsequent studies, that attention toward task relevant cues is enhanced as arousal increases (i.e., at increased levels of physical exertion). However, as arousal increases beyond a zone of optimal functioning (individually and situationally effected) individual awareness of task relevant cues are not perceived (see figure 5(b)). It follows that for each situation, there exists an ideal arousal range that maximizes focus on task-relevant cues while blocking irrelevant cues. This arousal range will be person

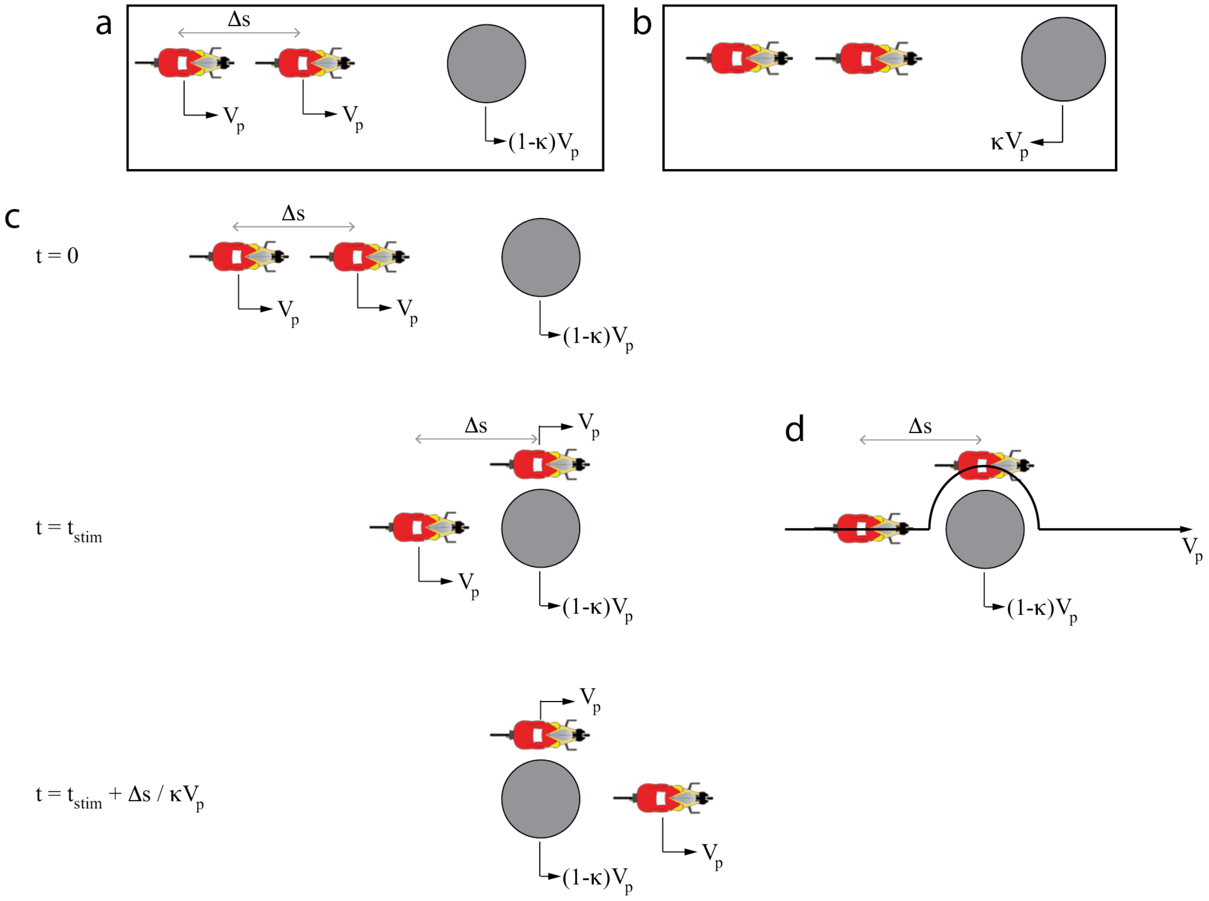

Figure S8. Interpretation of EOR cases with V_ϕ/V_c = constant (red markers in figure 3). **a,** This scenario can be interpreted as cyclists moving at the peloton speed V_p around a virtual obstacle, which is itself moving at a fraction $(1-\kappa)$ of the peloton speed (where $0 \leq \kappa \leq 1$). **b,** In the frame of reference of the peloton riders, the stimulus is moving towards them at velocity κV_p. **c,** In a characteristic transverse wave motion in this scenario, a leading rider moves laterally "around" the virtual obstacle. The following rider repeats this motion at a time $\Delta t = \Delta s/\kappa V_p$ later. Thus, the characteristic timescale associated with wave propagation increases linearly with spacing between riders. **d,** The scenario may be interpreted as a streamline in a fluid flow moving around a moving obstacle. The special case $\kappa = 1$ is instructive. Here, $\Delta t = \Delta s/V_p$, such that the characteristic wave propagation timescale is equal to the time it takes for each body to cover the space separating them. This is analogous to the situation observed when a streamline encounters a fixed obstacle in a fluid flow.

and task dependent. It has also been found that higher rates of intense exercise direct attention inward on the physical discomfort of acute exertion, additionally distracting the individual from relevant cues [6]. Therefore, it is important for athletes (cyclists) to be aware of their exertion rate, so that they can operate in their optimal zone of performance, focus on relevant task cues and filter out irrelevant cues, and continue to make ideal race decisions even as their arousal levels increase.

Furthermore, there have been studies that link intensity of physical exercise to brain metabolic processes and cognition. Some theories suggest there is a "loss of executive control functions during conditions of moderate to high-intensity exercise," as low level sensorimotor functions become prioritized [7, 8, 9], and "this pattern may be driven by a dynamic reallocation of brain metabolic resources from a frontal-parietal control network toward lower level salience networks [10] (see also [7, 8, 9]). One might expect this type of process to coincide with end of race (EOR) conditions in which a cyclist may physically be in an 'explosive' exertion and make no more executive decisions. Indeed, sprinters near the end of the race are lead out by other riders on their team going at near maximal levels of effort. The sprinters get themselves into the highly aroused state (but not too high, yet) to ensure that they can still think and keep wider fields of perception as the line approaches. Once they decide to 'explode' for the finish line and put themselves into the physical 'red zone,' their attention is most likely very narrow. One recent study found that "high-intensity exercise induced arousal states...accentuated central detail memory" [11]. Therefore, sprinters can recall race sprints in detail related to what they perceived and what they did, while happening at high levels of arousal. This study also suggests its data were tending toward indicating that *peripheral* detail memory was decreased during exercise.

There is most likely a learning curve such that by the time a cyclist competes at the Tour de France, they are no longer affected by expected race events. The final sprint is not as stressful

for the sprinter because they have become adept at handling the expected nature of the finishing dynamics. Professional cyclists have often developed coping techniques or strategies to mitigate the higher rates of arousal. Brunyé and Mahoney state that "theories of arousal-based competition suggest that arousal during encoding (whether due to induced arousal or presented stimuli) selectively influences memory for high priority, salient information" [11] (see also [12]).

5 Overview of the Tour de France professional bicycle race

Over the course of 21 daily stages, throughout the month of July, the Tour de France (TdF) captures the attention of cycling fans around the world. Twenty-two teams of eight riders compete within each stage for cumulative individual classifications throughout the competition, but most eyes focus on the prestigious yellow jersey (maillot jaune), the iconic apparel worn by the current overall race leader. Each team is composed of riders with varying specialties, and in each stage, teams decide their plan for the day, utilizing individuals' expertise to optimize the team's collective performance towards those goals. While this paper argues that the moment by moment dynamics in the cycling peloton are governed primarily by the human visual sensory system, longer timescale dynamics are governed by individual and team objectives. Teams with potential winners of the overall race (determined by lowest cumulative time), tend to expend a lot of energy riding at the front of the peloton to pace, shield and protect their team leader. Teams with expert sprinters, or strong climbers, rally around those individuals with primary objectives of winning individual daily stages. Team objectives can change mid-race; e.g, if a team leader is injured and cannot continue, then the team objectives will shift. Each team's goals and daily objectives are directed by one sporting director (directeur sportif), an individual who manages the intricacies of the team in training and on the course. While riders can be on the bike for upwards of six hours on a given day, it is the director's job to develop, implement,

and adapt their team's plan and communicate that to the riders on the course.

The range of team goals (overall cumulative victory, individual stage victory, climbing champion, etc.) mean that teams have different incentives, and there are numerous "races within the race". The daily stages are varied to accentuate these different objectives. The 2016 TdF analyzed herein consisted of 9 *flat* stages, 10 *mountain* stages and 2 *individual time trial (ITT)* stages [13]. The overall winner was Christopher Froome with a cumulative time of 89 hours, 04 minutes, 48 seconds; the second place rider was 4 minutes, 05 seconds behind. The flat stages are considered to be "sprinter" stages, which are characterized by a fast paced, highly contested end of race condition. For stages that are considered key for the overall competition, top riders will watch each other and adapt tactics based on each other but can afford to ignore small groups of breakaway riders that compete for that day's stage, as they are far enough down the cumulative time competition so as not to be a substantial threat. To be successful, sprinters will be of a heavier body type, and can struggle on hilly terrain, frequently finishing mountain stages over 15 minutes behind the stage winner. The high speeds of the race mean that racing cyclists generally need to ride as a group (the peloton) to benefit from aerodynamic drafting. In the ITT stages, cyclists ride individually without the benefit of drafting. As a result, the largest time gaps in cumulative time tend to be established on mountain and ITT stages. However, for the vast majority of the race, cyclists ride within large pelotons.

References and Notes

[1] Kalal, Z., Mikolajczyk, K., Matas, J. *et al.* Tracking-learning-detection. *IEEE transactions on pattern analysis and machine intelligence* **34**, 1409 (2012).

[2] Hartley, R. & Zisserman, A. *Multiple view geometry in computer vision* (Cambridge university press, 2003).

[3] Wilson, D. G., Papadopoulos, J. & Whitt, F. R. *Bicycling science* (MIT press, 2004).

[4] Meijaard, J. P., Papadopoulos, J. M., Ruina, A. & Schwab, A. L. Linearized dynamics equations for the balance and steer of a bicycle: a benchmark and review. In *Proceedings of the Royal Society of London A: Mathematical, Physical and Engineering Sciences*, vol. 463, 1955–1982 (The Royal Society, 2007).

[5] Easterbrook, J. A. The effect of emotion on cue utilization and the organization of behavior. *Psychological review* **66**, 183 (1959).

[6] Hutchinson, J. C. & Tenenbaum, G. Attention focus during physical effort: The mediating role of task intensity. *Psychology of Sport and Exercise* **8**, 233–245 (2007).

[7] Chang, Y.-K., Labban, J., Gapin, J. & Etnier, J. L. The effects of acute exercise on cognitive performance: a meta-analysis. *Brain research* **1453**, 87–101 (2012).

[8] Dietrich, A. Transient hypofrontality as a mechanism for the psychological effects of exercise. *Psychiatry research* **145**, 79–83 (2006).

[9] McMorris, T. *Exercise-cognition interaction: Neuroscience perspectives* (Academic Press, 2015).

[10] Elton, A. & Gao, W. Divergent task-dependent functional connectivity of executive control and salience networks. *Cortex* **51**, 56–66 (2014).

[11] Brunyé, T. T. & Mahoney, C. R. Exercise-induced physiological arousal biases attention toward threatening scene details. *Psychological reports* 0033294117750629 (2018).

[12] Mather, M. & Sutherland, M. R. Arousal-biased competition in perception and memory. *Perspectives on psychological science* **6**, 114–133 (2011).

[13] Tour de france 2016. `http://www.cyclingnews.com/races/tour-de-france-2016/`. Accessed: 2018-11-30.
